# Diagnosis of *Schistosoma mansoni* infections: what are the choices in Brazilian low-endemic areas?

**DOI:** 10.1590/0074-02760180478

**Published:** 2019-03-28

**Authors:** Vanessa Silva-Moraes, Lisa M Shollenberger, Liliane Maria Vidal Siqueira, William Castro-Borges, Donald A Harn, Rafaella Fortini Queiroz e Grenfell, Ana Lucia Teles Rabello, Paulo Marcos Zech Coelho

**Affiliations:** 1 Fundação Oswaldo Cruz-Fiocruz, Instituto René Rachou, Biologia do Schistosoma mansoni e sua interação com o hospedeiro, Belo Horizonte, MG, Brasil; 2 Fundação Oswaldo Cruz-Fiocruz, Instituto René Rachou, Grupo de Pesquisas Clínicas e Políticas Públicas em Doenças Infecciosas e Parasitárias, Belo Horizonte, MG, Brasil; 3 Universidade Federal de Ouro Preto, Laboratório de Enzimologia e Proteômica, Ouro Preto, MG, Brasil; 4 University of Georgia, College of Veterinary Medicine, Department of Infectious Diseases, Athens, GA, United States of America; 5 Old Dominion University, Department of Biological Sciences, Norfolk, VA, United States of America

**Keywords:** Schistosoma mansoni, low-endemic area, diagnosis, immunoassay, POC-CCA

## Abstract

The population of Brazil is currently characterised by many individuals harbouring low-intensity *Schistosoma mansoni* infections. The Kato-Katz technique is the diagnostic method recommended by the World Health Organization (WHO) to assess these infections, but this method is not sensitive enough in the context of low egg excretion. In this regard, potential alternatives are being employed to overcome the limits of the Kato-Katz technique. In the present review, we evaluated the performance of parasitological and immunological approaches adopted in Brazilian areas. Currently, the diagnostic choices involve a combination of strategies, including the utilisation of antibody methods to screen individuals and then subsequent confirmation of positive cases by intensive parasitological investigations.

Schistosomiasis is one of seventeen neglected tropical diseases (NTDs) listed by the World Health Organization (WHO), and this disease presents a substantial public health and economic burden and is considered a disease of poverty. An estimated 779 million people are at risk of infection, and approximately 252 million people are currently infected.^1^ ^,^ ^2^ The Global Health Estimates of 2015 attributed 3.51 million disability-adjusted life years (DALYs) and 10.1 million deaths in 2016 to schistosomiasis, which is a mortality figure that has been challenged as a gross underestimate.^3^ ^,^ ^4^ In the Americas, the only known species of parasite that is associated with intestinal schistosomiasis and continues to be endemic in parts of Brazil, Venezuela, and the Caribbean is *Schistosoma mansoni*.^5^

Schistosomiasis induces acute, severe, and chronic morbidity among those who are infected. If untreated, it can result in anaemia, splenomegaly, and fibrosis that can cause portal hypertension, esophageal varices, and upper gastrointestinal bleeding; in serious cases, it triggers kidney and neurological complications and death.^6^ Three major control strategies have been adopted to combat schistosomiasis, and these include chemotherapy, diagnostics, and improvements in sanitation and hygiene.^7^ Since the mid-1980s, the main global strategy to fight schistosomiasis has been treatment with praziquantel (PZQ).^8^ PZQ treatment in general has reduced the high levels of prevalence and intensity of infection so that control programs are able to manage morbidity. After decades of extensive PZQ use, however, low-intensity infections exhibiting high re-infection rates remain and transmission persists.^9^ ^,^ ^10^

The failure of PZQ to eliminate schistosomiasis has placed new emphasis on the role of diagnostics in the control of schistosomiasis. The plan for 2020 that focuses on “elimination of disease as a public health problem” also now emphasises attainable strategies for accurately diagnosing low-intensity infections.^11^ ^,^ ^12^ ^,^ ^13^ As a result of intensive control strategies, many previously high-endemic areas are now considered low-endemic areas. They are characterised by < 10% prevalence, and the majority of infected individuals harbour low-intensity infections (number of eggs per gram of faeces (EPG) is < 100).^9^ ^,^ ^14^ ^,^ ^15^ Detection of schistosome eggs in stools by microscopic examination using the Kato-Katz technique (K-K) is the recommended method by the WHO for diagnosing an active infection. K-K, however, possesses poor sensitivity for low-intensity infections and therefore cannot be currently considered the gold standard.^16^ ^,^ ^17^ Improvements in the diagnostic field are strongly supported by control programs in Brazil, which is a low-endemic country where PZQ mass drug administration (MDA) is only indicated in localities with egg-positivity above 25% and the main control strategy focuses on strengthening diagnoses and treatment of infected individuals at the primary care level.^5^ ^,^ ^18^ ^,^ ^19^ ^,^ ^20^

Over the past 40 years, Brazil has developed an extensive history regarding the fight against schistosomiasis. Integrated control measures, such as investments in basic sanitation and hygiene, improvement in the population’s income levels and quality of life, and chemotherapy have yielded considerable success in terms of reducing the prevalence, transmission, and morbidity.^15^ The number of severe clinical cases and deaths has decreased significantly.^21^ Currently, Brazil has chronic patients living in endemic areas, as well as acute cases derived from migration of populations due to urbanisation and rural tourism.^18^ ^,^ ^22^ ^,^ ^23^ ^,^ ^24^ ^,^ ^25^ ^,^ ^26^ ^,^ ^27^ ^,^ ^28^ ^,^ ^29^ Low parasite loads are persistent among most individuals from endemic areas as a result of consecutive rounds of treatment and frequent exposure to the infective agent.^18^ ^,^ ^30^ Further, low-intensity infections may occur upon a single exposure to the infective agent, such as exposure during tourism to transmission areas and movement of residents from endemic areas to urban centres.^27^ ^,^ ^28^ ^,^ ^29^

The prevalence in Brazil was estimated at 1% by the National Schistosomiasis and Soil-transmitted Helminth Infection Survey (INPEG) conducted between 2010 and 2015.^15^ When comparing the two last surveys, a large reduction in prevalence was observed, (10.09% between 1949 and 1953 and 9.24% between 1975 and 1979);^15^ however, infection persists, and the lack of a diagnostic method that is compatible with the epidemiological scenario presents a concern.^13^ ^,^ ^15^ ^,^ ^17^ Although K-K is affordable and suitable for low-income areas, its sensitivity decreases dramatically in the current conditions of low egg excretion, and it is not as efficient for determining disease prevalence as it was in the past when medium and high parasite loads (> 100 EPG) were predominant.^26^ Infected individuals who are not diagnosed correctly and do not receive the proper treatment remain infected and contribute to the maintenance of transmission and the establishment of a new focus.^18^ ^,^ ^31^ ^,^ ^32^

The INPEG was the major survey that covered all states within the federation of Brazil. A total of 197,564 school-age individuals (7-17 years old) were evaluated in 27 states by an analysis of parasitological method (two K-K slides). Studies in Brazilian areas, however, have recently demonstrated that disease prevalence has been underestimated by a factor of between two and four, likely due to the inability of the K-K to detect low-intensity infections.^18^ ^,^ ^32^ ^,^ ^33^ This failure to determine the accurate disease prevalence led to shortcomings in decisions made during national control programs, and this elevated prevalence remains a major challenge for disease elimination.^26^ Given this, the question arises as to whether the low prevalence rate observed indicates that Brazil is moving toward elimination, or whether Brazil is simply underestimating prevalence because of the use of a method that is not sensitive enough to detect low-intensity infections. If the goal of elimination is a priority for the WHO, new and more sensitive methods must be utilised to achieve this purpose.

Significant progress has been made in regard to development of more sensitive tests. Diagnoses using antibodies,^24^ antigens,^34^ and DNA^18^ ^,^ ^35^ have been evaluated. These techniques have exhibited high sensitivity, however, their reduced specificity in comparison to microscopy-based methods make them inadequate as single-use tests.^33^ ^,^ ^36^ Because of the high specificity of egg-based techniques for detecting active infections, new sensitive parasitological methods have been developed, including the saline gradient (SG)^37^ and Helmintex (HX).^38^ Improvements have also been made to the conventional protocol of K-K, and these include increasing the number of samples and the number of slides to be evaluated.^18^ ^,^ ^31^ ^,^ ^39^

As an alternative to time-consuming parasitological methods, a rapid diagnostic test (RDT) based on the detection of active parasite-secreted antigen in urine was also developed. The point-of-care circulating cathodic antigen test (POC-CCA®) has been commercially available since 2008, and owing to its higher sensitivity than K-K, it has been suggested as a suitable substitute for K-K in *S. mansoni* prevalence mapping.^40^ ^,^ ^41^ ^,^ ^42^ ^,^ ^43^ ^,^ ^44^ ^,^ ^45^ The majority of studies validating POC-CCA®, however, were conducted in Africa using K-K as a reference method.^45^ As K-K is not sensitive enough to detect low-intensity infections and cannot be considered the gold standard for evaluation of new methods, POC-CCA® performance in low-endemic areas remains to be validated before it is released for general use.^46^ Only 10 studies were conducted in Brazil, which has a significantly different prevalence and morbidity profile, and these tests yielded controversial results regarding sensitivity and specificity.^25^ ^,^ ^26^ ^,^ ^33^ ^,^ ^36^ ^,^ ^39^ ^,^ ^47^ ^,^ ^48^ ^,^ ^49^ ^,^ ^50^ ^,^ ^51^

As human schistosomiasis is becoming more of a low-endemic area disease, and the WHO-recommended method K-K has low efficiency for accurately detecting low-intensity infections, certain strategies have been adopted to overcome the current limitations. We review here some strategies that are applied in Brazil, which is a low-endemic country with hard-to-detect individuals targeted to achieve elimination. Our approach focuses on laboratory and field-based parasitological and immunological assays that can be summarised in three steps. The first is improvement of parasitological methods (increased number of samples or K-K slides and addition of other more sensitive egg-based assays), the second is antibody-based detection as an auxiliary tool to parasitological investigations (acute diagnostic and preliminary screening in endemic areas), and the third is antigen-based RDT POC-CCA® as a possible candidate to be part of the control. These methods have been used alone or in combination and have been accepted because of their easy application and accessible costs.

It is important to emphasise that molecular techniques have also been applied in addition to parasitological and immunological methods in Brazilian endemic areas with significant performance.^18^ ^,^ ^35^ ^,^ ^52^ ^,^ ^53^ ^,^ ^54^ ^,^ ^55^ They have been described as a complementary tool for parasitological methods for detecting low burden individuals and during assessment of cure after treatment.^18^ ^,^ ^52^ ^,^ ^56^ Polymerase chain reaction (PCR)-based detection of parasite DNA in stools or urine is more sensitive than parasitological methods and has been employed increasingly for diagnosis in high-resource settings;^57^ however, the infrastructure needed and the costs of reagents and equipment remain relatively high, which limits its use in low-resource settings such as Brazil. Some authors have estimated the costs as US $6 and US $8 for conventional and real-time PCR, respectively,^14^ and US $17 for PCR-enzyme-linked immunosorbent assay (PCR-ELISA).^53)^

## The analyses of multiple slides and a combination of parasitological techniques increases the diagnoses of low-intensity infections

The greatest value of parasitological methods is also the most significant challenge in the development of new diagnostic tools. Specifically, this is the ability to count eggs to accurately determine the intensity of infection.^58^ ^,^ ^59^ In Brazilian programs, mapping, estimating the global burden of disease, evaluating anti-schistosomal drug efficacy, monitoring of control programs, and verification of local elimination all depend on accurate diagnoses that are directly related to parasitic load.^5^ ^,^ ^17^ ^,^ ^19^

The K-K method is based on quantification of faecal eggs and exhibits a high level of specificity.^16^ It is simple (i.e., requires minimal laboratory equipment and a well-trained laboratory technician), less laborious than many other procedures, inexpensive (approximately US $0.2 per sample), and can be used under field conditions.^20^ K-K slides are prepared by using standardised 41.7 mg templates from which eggs are counted, and after multiplication by a factor of 24, this analysis reveals an estimate of eggs per 1 g of stool.^16^ Two K-K slides are recommended for active-search surveys because of their operational cost-effectiveness.^17^ This examination protocol is suitable for moderate (100-399 EPG) and heavy (> 400 EPG) intensity infections; however, two K-K slides fail for low-intensity infections (< 100 EPG), as more faecal matter is required.^18^ ^,^ ^32^ Several studies have investigated the effect of modifications to the K-K protocol, such as collecting stool samples on different days, increasing sample size and the number of slides analysed, and the addition of other parasitological methods in low-endemic areas.^18^ ^,^ ^31^ ^,^ ^32^ ^,^ ^33^ ^,^ ^36^ ^,^ ^60^ ^,^ ^61^

In one study, Rabello et al.^60^ suggested that K-K from three faecal samples (two slides each) collected on different days functions better in low-endemic areas. In this study, an analysis of three samples provided higher sensitivity compared to one or two samples. Enk et al.^32^ also demonstrated a direct relationship between the number of slides analysed in different faecal samples and the rate of positive infection. The prevalence of disease detected from one K-K slide from one faecal sample compared to six K-K slides from three faecal samples increased from 13.8% to 25.9%. Additionally, when K-K was combined with the formol-ether centrifugation technique, the prevalence increased to 35.4%. These modifications resulted in higher positive test rates and indicated that individuals required treatment, and the findings would have otherwise been missed if only a single sample was used. These modifications, therefore, enhance the efficiency of schistosomiasis control programs in low-endemic areas.^32^

Testing faecal samples that were collected on different days provides a significant strategy for improving detection owing to day-to-day variation in egg output. In the field, however, it is clearly more practical to analyse a single sample by additional slides and techniques due to a lack of patient compliance. Siqueira et al.^18^ ^,^ ^31^ performed an extensive evaluation based on an in-depth diagnostic survey and follow-up after treatment in endemic areas in the northern Minas Gerais state. Their results demonstrated the importance of simply increasing the number of slides to increase detection, regardless of the number of samples. The positivity rate from two K-K slides was 8% compared to 14.4% when 12 K-K slides were read for one stool sample. The 12 K-K protocol was later employed to determine 100% cure rate after 30 days of treatment. When a total of 18 K-K slides were combined with the commercial TF-Test kit, the positivity rate increased to 35.8%, a 4.5-fold increase relative to two K-K slides. The TF-Test that was described by Gomes et al.^62^ is a commercial technique that is based on the centrifugation-sedimentation of three faecal samples collected on different days using ethyl acetate as a reagent. Several authors have evaluated the performance of this technique in the context of intestinal parasite diagnosis. Carvalho et al.^63^ demonstrated efficient detection of protozoa cysts and other geo-helminths by the TF-Test. Nacife et al.^64^ and Siqueira et al.^31^ showed that for schistosomiasis, this technique did not demonstrate effective diagnostic performance; however, the combination of TF-Test with the K-K technique increases the overall sensitivity.

The addition of multiple parasitological methods for analysing a single sample has demonstrated advantages for detecting low-burden individuals. The SG is a non-sophisticated but highly sensitive technique that uses 1 g of faeces (two procedures × 500 mg = 1000 mg) from a single sample.^37^ This protocol is low-cost (US $1 per sample) and is similar to the 24 K-K slides approach (24 × 41.7 mg = 1000 mg). During this process, the suspension of faeces in 0.9% saline is subjected to a slow flow of 3% saline for 1 h. The gradient variation allows the eggs to sediment to the bottom. The sediment (1-2 mL) is removed and placed on microscope slides to identify and quantify *S. mansoni* eggs.^37^ The advantage of SG is that a number of samples can be performed at the same simultaneously. With a device that has 12 separating columns, it is possible to run six different samples for 1 h, which is equivalent to 144 K-K slides. Another advantage of SG is the smaller number of slides needed for analysis, which totals 40 min of reading per sample, while 24 K-K slides require 90 min for reading.^37^

The SG has been used in addition to the K-K technique with increased detection of cases resulting from increased sensitivity by analysis of 2 g of faeces from one single sample. As the number of slides from these combined techniques is high, it is feasible in populations no greater than 350 individuals, as the slides are prepared in the field and brought to the laboratory for analysis. Siqueira et al.^39^ evaluated an area of 141 individuals by combining 24 K-K slides from a single sample with two SG procedures resulting in 24.1% prevalence, while two K-K slides estimated only 10.6% prevalence. Further, this improved combination of techniques was considered an adequate reference standard to determine the performance of POC-CCA®, which reached a 22.7% positivity rate.

The HX is a parasitological method that uses up to 30 g of faeces from a single sample. The methodology is based on the use of magnetic beads to trap eggs in a magnetic field.^38^ The high amount of examined faecal matter (714-fold higher than K-K) increases the probability of finding small numbers of eggs, making it a highly sensitive egg-based method.^33^ ^,^ ^36^ ^,^ ^65^ HX has been described as more sensitive than the K-K and SG methods in conditions of low egg excretion. Teixeira et al.^38^ demonstrated 100% sensitivity for egg burdens higher than 1.3 EPG. In a study by Caldeira et al.,^65^ HX detected 11/77 individuals while K-K detected 7/77 in a Brazilian low endemic area. Pinheiro et al.^61^ observed that HX performed better in determining prevalence (47.1%) than did K-K (8.75%) or SG (18%). Oliveira et al.^33^ supported the previous observations in their study where HX exhibited 84% sensitivity compared to 41% from two K-K slides and 45% from SG. Additionally, when all techniques were combined (18 K-K slides, SG and HX), the prevalence increased 2.3 times compared to conventional K-K.^33^ In a recent study by Lindholz et al.,^36^ HX was the most sensitive diagnostic method for schistosomiasis compared to POC-CCA® and two K-K slides. HX demonstrated 100% sensitivity for identifying low-intensity infections, most which were characterised by less than one EPG. Because of its high performance, HX was considered a gold standard to be used as a reference method during evaluation of new diagnostic techniques.^36^

Although sensitive, the applicability for large-scale screening in the field remains a disadvantage. The requirement of a large sample size and time-consuming sieving and sedimentation processes (approximately 3 h) make it complex and labour-intensive.^36^ ^,^ ^38^ Major efforts have been devoted to applying HX to field applications while reducing the costs (US $3 per sample).^36^ Some adaptations involve reducing the time spent screening sediment samples via addition of detergent during concentration steps and also staining the final sediment prior to microscopic evaluation.^66^ While the use of HX as a routine diagnostic method for schistosomiasis still requires further optimisation, its use can be recommended in efforts to eliminate schistosomiasis in low-endemic areas. As HX constitutes an accurate diagnostic method, it can be applied in the field for cure control, as a certification criterion to interrupt transmission, and also in addition to other methods as a final step in a sequential screening algorithm.^65^ ^,^ ^66^ Further, in the era of validation of new diagnostic methods, HX can assume the role of a reference method for comparative performance.^33^ ^,^ ^36^ ^,^ ^66^

While studies have shown that increasing the number of slides analysed in single or multiple samples in combination with other diagnostic assays increases sensitivity, it can be argued that the additional sampling and methods make the improved diagnostic procedure impractical for use in the field and not economically viable. To address this point, it is important to analyse the economic cost-benefit. For example, the standard two K-K (41.7 mg of faeces) costs US $0.4 and requires 8 min to read. Increasing to 24 K-K slides (1000 mg of faeces) increases the cost to US $4.8 and requires 90 min to analyse. Alternatively, analysing 1000 mg of faeces by SG costs US $1 and requires 40 min to read. For hard-to-detect cases, HX is the most sensitive with a cost of US $3 and 3 h of development.^36^ Thus, improved diagnostic methods cost more and require more time than the K-K method (two slides); however, if undiagnosed egg-positive individuals remain infected, they will continue to contribute to disease transmission and potentially establish new areas of infection. This would require additional resources to be utilised for continued diagnosis due to an inability to reduce prevalence, as all positive individuals are not being treated. Further, undiagnosed individuals may develop various chronic diseases related to schistosome infection, including disabilities, all of which increase health care costs.^67^ Thus, in low-prevalence settings, the increased sensitivity and reduction of infection-related costs are an economically viable approach compared to the need to perform consecutive tests without any increase in sensitivity. This ultimately increases time and costs while not providing a significant impact on reductions in disease prevalence or human morbidity.

Overall, control programs that can obtain sensitivity levels near real prevalence in low-endemic areas would employ 24 K-K slides in combination with two SG procedures for analysing 2 g of faeces from a single sample. Such large-scale evaluation limits the operational advantages of this protocol, as greater fieldwork is required. These techniques are, however, practical in small communities (350 individuals) with high patient adherence. Utilising methods that are independent from laboratory infrastructure is a considerable advantage, as the highest infection and morbidity rates for the disease occur in the poorest and least developed regions.^(18,24,25, 26,31,39,68)^

## Antibody detection is a valuable tool to identify egg-negative infections

Antibody-based methods can detect positive reactivity in K-K-negative individuals. As antibodies to the parasite develop during the first weeks after infection, they can be detected before eggs that yield higher sensitivities.^69^ ^,^ ^70^ In Brazil, antibody-based assays have been described in acute individuals from non-endemic areas that were recently exposed and combined with parasitological techniques during epidemiological surveys.^14^ ^,^ ^23^ ^,^ ^24^ ^,^ ^29^ ^,^ ^35^ ^,^ ^71^ ^,^ ^72^ ^,^ ^73^

Low-intensity infections may occur among individuals after a single exposure to endemic areas, and these individuals include tourists and those in travellers’ medicine clinics.^74^ These individuals may develop acute infections that are asymptomatic or display unspecific symptoms, and given this, clinical examination and egg microscopy alone may not detect infection/exposure.^23^ ^,^ ^29^ ^,^ ^75^ In clinical practice, positive serology in K-K-negative individuals from non-endemic countries who have been exposed to schistosomiasis-endemic areas is usually sufficient to prescribe treatment with PZQ.^74^

In recent years, a new pattern of schistosomiasis transmission has been described in Brazil. It is related to recreational activities associated with rural or ecological tourism, migratory flows, such as urbanisation, and accompanying changes in social dynamics.^22^ ^,^ ^27^ ^,^ ^76^ Although the disease is usually thought of as a rural problem, it is increasing in large urban centres of Brazil due to migration of residents from endemic rural areas.^28^ Lambertucci et al.^29^ described an atypical outbreak in a non-endemic area of Brazil due to the migration of infected workers from a schistosome-endemic area. Transmission occurred when tourists swam in a natural pool recently infected by the migrant workers, which became a new focus of transmission. As these tourists had no history of having visited endemic settings and likely had no eggs in the faeces, the presence of antibodies could suggest an active infection. Among the 50 tourists examined, only 38% individuals were egg-positive in K-K. When ELISA for detecting IgG using schistosomula antigen (SmTeg) was performed, 80% of the tourists were positive.^23^ Further, incorporation of multi-isotype detection (IgG/A/M/E) makes these tests potentially more sensitive for the detection of schistosomiasis during pre-patent and acute phases. Previous studies have shown that detection of IgA against soluble egg antigens (SEA) and IgA/IgM against soluble worm antigens (SWAP) via ELISA are good alternatives for identifying acute infection.^77^ ^,^ ^78^ ^,^ ^79^

Diagnostics based on detection of antibodies perform differently in individual versus community-based settings. Residents in endemic areas produce antibodies by regular and frequent exposure to the infective agent, but this does not mean that they are infected. Similarly, previously infected residents retain circulating antibodies for a long period of time after PZQ treatment. Studies demonstrate that antibody levels begin to decrease after approximately 6 months of treatment but are still detected two years later or longer.^70^ ^,^ ^80^ In individuals living in endemic areas, it is not clear if the persistence of IgG (negative parasitological) reactivity after treatment is due to reinfection, non-response to chemotherapy, or natural resistance to disease.^70^ ^,^ ^81^ ^,^ ^82^ Given these limitations, antibody-based detection methods are not currently the first choice for endemic areas; however, the determination of immunoreactivity against different schistosome antigens can identify infections with loads as low as 1 EPG.^24^ ^,^ ^68^ ^,^ ^83^ ^,^ ^84^ ^,^ ^85^ ^,^ ^86^ This fact had led to the introduction of these methods as a complementary tool during epidemiological surveys in low-endemic areas. Its use decreases false-negative cases seen when few K-K slides are applied in conditions of low egg excretion.^24^ ^,^ ^35^ ^,^ ^73^ ^,^ ^87^ ^,^ ^88^ ^,^ ^89^

Detection of antibodies by ELISA has demonstrated a good correlation between parasitological methods when compared to a reference method in low-endemic areas in Brazil. IgG-ELISA-SWAP exhibited 90% sensitivity/specificity and a Kappa index of 0.85 when compared to 18 K-K slides.^24^ The study by Oliveira et al.^90^ demonstrated 98% sensitivity and 97.7% specificity for IgM-ELISA. Both studies identified low burden individuals misdiagnosed with one K-K slide. Even though the high performance of immunoassays indicates them as alternatives to the K-K, they are not optimised for single use tests. Conversely, they are considered potential screening tests that can lead to accurate diagnoses and informed treatment decisions.

In previous studies, after the first diagnostic round by ELISA and K-K, the IgG-positive but egg-negative individuals submitted two additional stool samples (second and third samples). Most IgG-positive, but egg-negative patients became egg-positive (increased 6% to 10% positivity rate) and were then treated with PZQ.^87^ ^,^ ^88^ In the study by da Frota et al.,^89^ after an initial screening, 85 IgG-positive, but egg-negative cases were evaluated a second time. After additional sampling and preparation of more K-K slides, the positivity increased from 3.8% to 8.7%. The ELISA detected 96/287 individuals with no false-negative results (100% sensitivity, 72.9% specificity). Pinheiro et al.^61^ adopted IgG-ELISA as a screening test in an endemic area in Brazil. Only individuals positive for ELISA were submitted for parasitological examination by combined K-K (three slides), SG, and HX. From 33 initial IgG-positive cases that provided faecal matter for analyses, 22 were positive. If three K-K slides were used individually, only three individuals would have been diagnosed and treated. Espírito-Santo et al.,^35^ using IgM-ELISA and IgG-ELISA, detected antibodies in 21.4% and 11.6%, respectively, while two K-K slides showed 0.7% positivity. Because of no false-negative results, the authors suggested an algorithm composed of screening with a combined IgM/IgG-ELISA, a parasitological evaluation to confirm IgG/IgM-positives, and molecular PCR for persistent egg-negatives.^35^

Owing to the high sensitivity of ELISA and consequently less frequency of false-negative results, these studies indicated that ELISA may be useful for screening populations. Although ELISA is superior to K-K in preventing false-negative results and thus increases the chance of not missing infected individuals, the test led to a large-number of false-positive results.^24^ At this point, it is important to emphasise that subsequent confirmation of diagnoses by egg-based methods or other more sensitive, but highly specific, tests is necessary. The single use of ELISA can lead to significant over-treatment, but its combination with parasitological methods reduces time consumption during field work. The possibility of automation and analysis of a large number of samples simultaneously are more rapid than coproscopy and enable primary screening and selection for more robust evaluation.

The antigens used in immunoassays are crucial for sensitivity and specificity testing.^91^ In attempts to improve antigenic targets for antibody detection, several studies have been conducted using circulating antigens, purified fractions of the schistosomal life cycle, recombinant proteins, and synthetic peptides. The CCA was tested for the detection of antibodies by immunomagnetic separation (IMS) in a low-endemic area in Brazil by using four different targets, including crude CCA (CCAc), recombinant CCA (CCAr), and two peptides (CCApep1 and CCApep2). The IMS-CCAr yielded the best results with 100% sensitivity and 96% specificity without false-negative results. IMS-CCAc demonstrated good performance to access cure post-treatment (90% sensitivity and 92% specificity).^68^ The IgG-ELISA using recombinant 200 kDa tegumental protein (rSm200) demonstrated 90% sensitivity and 93.3% specificity with a strong correlation with egg burden in the same area.^86^ Another recombinant protein, SmRP26 (rSm22.3), was also used in ELISA in acute and chronic phases, with sensitivities of 83% and 32%, respectively, and a specificity of 97%.^92^ Pooled synthetic peptides were tested as antigens in ELISA using samples from Brazilian endemic individuals, and these provided a sensitivity of 86.8% and a specificity of 94.2%.^84^ Carvalho et al.,^85^ using computational tools, selected and produced seven synthetic peptides that were then tested in ELISA against endemic human sera and exhibited specificities of 46.15% to 100%, while sensitivities varied from 53.85% to 96.15%.

Among the studies discussed, high sensitivity and specificity were both observed for immunodiagnostic techniques compared to those observed using the parasitological reference method. One of most challenging tasks is attempting to evaluate new tests adopting the K-K performed with two slides as a gold standard.^45^ Because of its known limitations in populations with low-intensity infections, the use of K-K establishes inaccurate parameters during the validation process. Improvement of reference methods is required to elucidate the efficacy of new methods and their applicability at the community level. Latent class analysis (LCA) is a modelling process to estimate performance of diagnostic tests in the absence of a gold standard. Applying LCA enables straightforward assessment of the prevalence estimates from each test by comparing the distributions of estimated test and estimated infection prevalence.^48^ ^,^ ^93^ The introduction of statistical LCA is encouraged, as it yields more accurate sensitivities and specificities of new tests than those obtained by comparisons to an imperfect gold standard.^93^

Antibody-detection strategies have provided significant progress in the diagnostic field, but these strategies still possess limited applicability in differentiating active from past infection and determining the intensity of infection and success after treatment. Consequently, these cannot be used as a single test.^70^ ^,^ ^71^ ^,^ ^94^ As it is for use in Brazil, the diagnostic must exhibit high sensitivity and provide a low occurrence of false-negative cases to aid the goal of elimination. Combined serological and parasitological assays would improve diagnosis of low-intensity infections (acute or chronic cases) in both clinical and epidemiological studies. In acute cases, IgA/IgM/IgG-ELISA could provide alternatives to support the decision to treat in the absence of eggs in the stool. In populations at risk of infection, a preliminary screening using antibody-detection methods such as IgM/IgG-ELISA increases the sensitivity of investigation. A subsequent intensive parasitological examination (24 K-K slides plus two SG) can be performed to confirm positive cases, evaluate the intensity of *S. mansoni* infections, and identify the presence of other parasitic infections.

## Performance of circulating antigen-based RDT diagnosis in low-intensity infections

The highlight for antigen-based assays to detect low-intensity infections are high sensitivity compared to coprology and higher specificity compared to that of antibody-based assays.^95^ Direct detection of schistosome antigens in serum, urine, and other fluids has been considered as an alternative to egg-based methods. The majority of these methods have demonstrated the ability to discriminate active and cured (post-treatment) infection and a positive correlation between antigen capture and egg counts, suggesting an infection intensity determination. The circulating anodic antigen (CAA) and CCA have been the most investigated targets.^96^ As only live worms produce and release these antigens into the circulation, this makes CAA and CCA positivity a determinant of active infection. These genus-specific proteoglycan antigens of the schistosomal gut epithelium are released in the vomitus of worms and can be detected in serum and urine at approximately three weeks post-infection.^97^ ^,^ ^98^

Schistosome antigen detection methods were initially based on biochemical fractionation production of monoclonal antibodies.^99^ A study conducted in Brazil evaluated direct detection of CCA in serum obtained from low burden individuals using a capture monoclonal antibody. The IgG-ELISA yielded 92% and 100% sensitivity and specificity, respectively, equivalent to the analysis of 16 K-K slides from three faecal samples. IMS-CCA demonstrated significant correlations to egg counts (R^2^ = 0.99). The fluorescent IMS-CCA demonstrated 83% sensitivity post treatment.^34^ Because of their high quality performance, the immunoassays developed in laboratories were translated to rapid diagnostic tests (RDT), making these tests more applicable for field use. Currently, such tests are available as the up-converting phosphor-lateral flow circulating anodic antigen (UCP-LF CAA) and POC-CCA®.^41^ ^,^ ^100^ The ability to perform diagnostic assays using non-invasive samples, such as placing urine on an immunochromatographic strip, provides large-scale sample testing that requires only minimal infrastructure to assess the need for intervention in endemic areas.

The non-commercial UCP-LF CAA is a dry reagent format for quantitative detection of all *Schistosoma* species that exhibits excellent clinical sensitivity and specificity in urine and serum.^96^ ^,^ ^100^ The sample preparation requires treatment with TCA to release the CAA glycan target in the supernatant and subsequent pooling of samples using centrifugal 10 kDa filters.^101^ Previous studies have demonstrated the potential of UCP-LF CAA to detect a single worm pair, corresponding to an analytical sensitivity of < 0.1 pg/mL. Additionally, measurements of CAA in urine demonstrated a correlation to eggs counts from stool, suggesting a direct measure for worm burden.^100^ Only one trial was conducted in a low-endemic area in Brazil, and the results indicated that UCP-LF CAA was more sensitive than POC-CCA® (92% vs 17%) to detect low-intensity infections.^102^ UCP-LF CAA is a promising tool for low-intensity infections; however, it requires basic laboratory equipment (a micro-centrifuge and a thermo-shaker), and the UCP-LF strip reader makes it difficult to implement in a low resource field. Major efforts are being taken to adapt the assay to a completely field user-friendly format to bring it to market, which will allow POC applications as needed for test-and-treat approaches.^101^ ^,^ ^103^ ^,^ ^104^

The POC-CCA® has been commercially available since 2008, and it has been considered as a potential candidate for inclusion into schistosomiasis control programs.^105^ In contrast to UCP-LF CAA, POC-CCA® was only developed for urine testing, and there are large variations in the detection of other schistosome species. The ability to use non-invasive samples and to perform the assay on the spot followed by treatment without any additional steps, microscope, or highly-trained technician led to the rapid market growth of this test.^45^ ^,^ ^105^ The POC-CCA® has been widely used in Africa (low, moderate, and high endemic areas) for mapping, determining MDA, and assessing cure post-PZQ.^42^ ^,^ ^43^ ^,^ ^44^ ^,^ ^45^ ^,^ ^105^ ^,^ ^106^ ^,^ ^107^ ^,^ ^108^ Although it has been considered superior to K-K, its use as a single test to replace parasitological tests requires additional optimisation.^45^ ^,^ ^46^ The POC-CCA® performed poorly in several areas of Brazil. This is perhaps because of the different prevalence profiles observed in Africa, as the majority of the low-intensity infections in Brazil are denoted as < 25 EPG. There are 10 published studies that were conducted in Brazil.^25^ ^,^ ^26^ ^,^ ^33^ ^,^ ^36^ ^,^ ^39^ ^,^ ^47^ ^,^ ^48^ ^,^ ^49^ ^,^ ^50^ ^,^ ^51^ The decision to adopt POC-CCA® for use in low-endemic areas of Brazil remains questionable for a number of reasons. Specifically, inadequate estimation of sensitivity and specificity of the POC-CCA® exists because of the absence of a highly sensitive reference method to evaluate its performance, interpretation of trace as either a positive or a negative reading can occur, and this test possesses low sensitivity for the detection of low-intensity infections.

An appropriate reference method is necessary to evaluate the accuracy of POC-CCA® in low-endemic areas. Siqueira et al.^39^ adopted 24 K-K slides and two SG as reference method in their study. The sensitivity of POC-CCA® was 86.6% and 73.5%, and specificity was 84.6% and 93.5%, when compared to K-K (two slides) and the reference method, respectively. The study by Ferreira et al.^48^ demonstrated that the POC-CCA® sensitivity values were 64.3%, 61.1%, 58.3%, and 55.5%, and specificity values were 74.5%, 74.8%, 75%, and 75.6% when compared to those of one, two, four, and six K-K slides, respectively. The use of two K-K slides as a reference method overestimates the sensitivity of the POC-CCA® due to the low efficiency of K-K in detecting true positive individuals with low levels of infection.

Another important issue is how the sensitivity and specificity of POC-CCA® are influenced by the interpretation of trace as either a positive or a negative reading. Treating trace as positive is indicated in the manufacturer instructions for the commercial POC-CCA®, as this could represent low-intensity infections. In African low-endemic areas, even if trace readings are considered negative, the prevalence by POC-CCA® is uniformly higher than K-K.^45^ ^,^ ^107^ ^,^ ^109^ In Brazilian settings, however, this greatly increases the sensitivity and leads to substantially more false-positive cases. Silveira et al.^47^ demonstrated that sensitivity decreased from 85.4% to 68.7% and specificity increased from 78% to 97.6% when traces were considered negative compared to data obtained from K-K. Oliveira et al.^33^ showed a similar result in which the sensitivity decreased from 64.9% to 26.8% and the specificity increased from 69.2% to 98.3% compared to findings using the reference method.

Souza-Figueiredo et al.^110^ previously reported that POC-CCA® results are more in agreement with those of K-K when traces are considered negative in low-endemic areas. This is not considered in Brazilian studies, however, even if a reference method is adopted. Compared to the results given by K-K (two slides), Bezerra et al.^49^ demonstrated that agreement between POC-CCA® negative trace is higher than POC-CCA® positive trace (Kappa = 0.12 vs 0.03). Silva et al.^50^ presented divergent data from previous findings. Compared to the reference method, Lindholz et al.^36^ showed a higher agreement index when trace results were considered negative (Kappa = 0.15 vs 0.28), while Oliveira et al.^33^ demonstrated the opposite. Specifically, their results indicate better agreement when trace was interpreted as positive (Kappa = 0.34 vs 0.25). This discordance exists because of the tendency of POC-CCA® to estimate a high number of false negatives as a result of its poor ability to detect infection in extreme low-endemic areas. In three different studies from Brazil, regardless of if traces were considered positive or negative, about one third of the infected individuals exhibiting low-intensity infections (< 12 EPG) were misdiagnosed by POC-CCA®.^33^ ^,^ ^36^ ^,^ ^47^

Aiming to eliminate doubt concerning trace results and increase detection of low-intensity infections, Coelho et al.^111^ proposed an additional step for POC-CCA® in Brazilian endemic areas. The POC-CCA Lyo that uses 10-fold concentrated urine via lyophilisation improved conventional POC-CCA® sensitivity from 6% to 56% compared to sensitivity observed using a reference method (24 K-K slides plus two SG). After concentration, trace became positive in parasitological positive cases, but remained as trace in parasitological negative cases. Additionally, patients exhibiting 1-3 EPG, primarily determined as negative, became positive in POC-CCA Lyo. Considering trace results as negatives in POC-CCA Lyo, the agreement with the reference method was moderate (Kappa = 0.401); however, when considering trace as positive, the agreement was slight (Kappa = 0.125). The pattern from trace was well-defined as negative cases and low burden individuals were correctly diagnosed.

As the additional lyophilisation step is time-consuming (34 h) and the need of a lyophiliser makes it complex in the field, the POC-CCA FLT was subsequently developed. The POC-CCA FLT requires a 50 min concentration step using a 30 kDa filter, which eliminates the need for specialised technicians and only adds a small increase to the cost of conventional POC-CCA®. In a study by Grenfell et al.^26^ in the low-endemic northern Minas Gerais state, accuracy increased from 0.51 to 0.86 when POC-CCA® and POC-CCA FLT were compared to the reference method, respectively. Further, POC-CCA FLT was able to properly diagnose nearly 100% of the cases 30 days post treatment and agreed with egg examination. This tool was also evaluated in Samambaia, a socioeconomic and environmental condition that is similar to endemic areas, despite having only one individual egg-positive (eight EPG) among 99 residents. POC-CCA FLT diagnosed 94 of 99 as negative individuals (20 negative and 74 traces). If traces were considered as positives in conventional POC-CCA®, 63 individuals would be treated with PZQ unnecessarily. Thus, the interpretation of trace is a concern as it can provide an imperfect diagnostic criterion for treatment decisions.

The lack of correlation between visual band intensities and egg counts is one disadvantage discouraging the use of POC-CCA® in clinics. Intensity of infection (low, moderates and high) is based on egg counts following WHO guidelines.^112^ Adriko et al.^108^ and other studies in Africa demonstrated the association between the intensity of the coloration of the band and the intensity of *S. mansoni* infection according to EPG by the K-K method.^43^ ^,^ ^44^ ^,^ ^108^ In Brazilian settings, Oliveira et al.^33^ and Silveira et al.^47^ found a significant positive correlation between the scores of the POC-CCA® band and infection intensity classified by EPG according to the WHO. Grenfell et al.^26^ and Lindholz et al.^36^ did not observe this same correlation.

The Brazilian reports regarding POC-CCA® show divergent results. The highest sensitivity related to POC-CCA® is demonstrated when the WHO-recommended K-K (one-two slides) is used as a reference method and when traces are considered positives. When a combination of rigorous parasitological methods is applied as a reference method (multiple K-K slides, SG, and HX) during the evaluation of performance of POC-CCA®, sensitivity decreases and demonstrates misdiagnosis of a considerable proportion of low-intensity individuals. Considering traces to be positive also overestimates the sensitivity rate of POC-CCA®; however, only considering traces as negative was not enough to achieve adequate agreement with a reference method. The use of POC-CCA FLT that adopts trace as negative should be evaluated in additional endemic settings, as it demonstrated high accuracy compared to the reference method adopted. The concentration of urine step and the increased testing time from 20 min (conventional POC-CCA®) to 50 min (POC-CCA FLT) should not pose a problem for use in low resource settings.

## In conclusion

The question of what diagnostic choices are available in Brazilian low-endemic areas remains to be fully answered. In the absence of one single high-performance method, multi-integrated strategies may be more cost-effective in the short- and long-term to avoid missing infections. The development of highly accurate methods for all aspects of prevention, control, monitoring, and surveillance are reinforced continually and cannot be neglected. Multiple groups are working to develop the ideal diagnostic test (unique, high sensitivity and specificity, low cost, easy-to-perform, fast, field-format, etc.). Ideally, a new diagnostic test should offer characteristics in terms of accuracy and operational benefits to justify the transition from the currently adopted K-K (one-two slides). Unfortunately, no single test is yet available.

In all contexts, to make the best choice, it is crucial to adapt the diagnostic approach to the epidemiological situation, the current stage of control, and the population under investigation. The determination of infected carriers in Brazilian settings and low-endemic countries should involve the combination of various methods and potentially the definition of algorithms for accurately estimating the prevalence and the indicators used in the control programs and elimination plan.

Based on these studies, a potential strategy would be antibody screening owing to its high sensitivity and subsequent confirmation of positive cases with robust parasitological tests (24 K-K slides plus two SG). Such an approach will increase the overall sensitivity to obtain a more reliable value of disease prevalence. This in-depth parasitological examination has been conducted with success in five low-endemic localities in the Minas Gerais state and has provided an adequate reference method for the standardisation and validation of new diagnostic tools.^18^ ^,^ ^25^ ^,^ ^26^ ^,^ ^39^ The new version of POC-CCA FLT could be applied as a complementary tool to egg detection to evaluate its potential as a future substitute for multiple parasitological examinations. Further, to overcome the complexity of ELISA in the field, a second-generation of antibody-based RDTs has already been proposed that could be used together with detection of antigen in a multiplex strip on a reader.^96^
